# Concordance between sequential transbronchial lung cryobiopsy and surgical lung biopsy in patients with diffuse interstitial lung disease

**DOI:** 10.1186/s13000-019-0908-z

**Published:** 2019-12-04

**Authors:** Yoshiaki Zaizen, Yasuo Kohashi, Kishio Kuroda, Kazuhiro Tabata, Yuka Kitamura, Akira Hebisawa, Yuji Saito, Junya Fukuoka

**Affiliations:** 1 Department of Pathology, Nagasaki University Graduate School of Biomedical Sciences, 1-7-1 Sakamoto, Nagasaki, 852-8501 Japan; 2 Department of Respirology, HARUHI Respiratory Medical Hospital, Kiyosu, Japan; 3 Department of Clinical Pathology, Asahi Central Hospital, Asahi, Japan

**Keywords:** Interstitial lung disease, Cryobiopsy, Surgical lung biopsy, Pathology, Histological findings

## Abstract

**Background:**

Increasing evidence indicates the utility of transbronchial lung cryobiopsy (TBLC) for the diagnosis of interstitial lung disease (ILD). However, only one study has compared TBLC and surgical lung biopsy (SLB) performed on the same patients.

**Methods:**

We identified seven patients with ILD with TBLC and SLB. We evaluated the clinical characteristics and made a pathological diagnosis based on the official ATS/ERS/JRS/ALAT clinical practice guideline of idiopathic pulmonary fibrosis with both TBLC and SLB.

**Results:**

Six cases were diagnosed as Usual interstitial pneumonia (UIP) in both TBLC and SLB. One case was diagnosed as indeterminate for UIP with TBLC and probable UIP with SLB. Etiological diagnosis with TBLC and SLB were concordant in 2 cases of idiopathic pulmonary fibrosis (IPF) but discordant for other diagnoses. Major histological findings of UIP including dense fibrosis, peripheral distribution, and fibroblastic foci showed high concordance between TBLC and SLB, which implies that TBLC can reliably detect these features. In contrast, loose fibrosis, cellular infiltration, and airway disease showed poor concordance between the two methods.

**Conclusion:**

Our study showed that TBLC is useful for UIP diagnosis but not for other ILD. With a multidisciplinary approach, diagnosis of IPF may be determined by TBLC, whereas ILD other than IPF may require SLB.

## Summary at a Glance

We compared pathology diagnosis and histological findings of cryobiopsy and surgical lung biopsy from identical patients in detail and found that cryobiopsy is useful for the recognition of UIP pattern, but may be cautious for other diseases. Findings such as organizing pneumonia may not be represented.

## Background

Transbronchial cryobiopsy (TBLC) is a relatively new method for obtaining lung tissue for diagnostic purposes [1]. It has been performed mainly for the diagnosis of lung endobronchial neoplasms and their genetic testing [2, 3]. In recent years, it has also been used for the pathological diagnosis of diffuse lung disease [4]. The method can be used to collect approximately 10–30 mm^2^ of tissue samples which often cover enough areas to observe the primary lobules of the lung parenchyma [1, 5]. This is larger than the specimen collected using forceps biopsy [6]. These samples include relatively less artifacts such as nuclear crush and alveolar collapse than samples from transbronchial forceps biopsy and provide more information needed for making a pathological diagnosis of diffuse lung disease [7, 8]. In past studies, TBLC was diagnostic for diffuse lung disease in 70–80% of cases [4, 9–12], which is higher than transbronchial lung forceps biopsy [5, 13]. TBLC is accurate and efficient especially in diagnosing Usual interstitial pneumonia (UIP) [14]. In addition, similar to surgical lung biopsy (SLB), improvement in diagnostic accuracy has been reported by multidisciplinary discussion (MDD) when making a diagnosis with TBLC samples [15, 16]. However, most studies did not compare TBLC and SLB in the same patients. Observational differences between TBLC and SLB caused by different sample sites and methods are important information in diagnostic confirmation. Recently, Romagnoli and Colby reported poor concordance of pathology diagnoses between TBLC and SLB [17]. However, at present, no study has performed TBLC and SLB on the same patients and compared their histopathological characteristics.

## Methods

### Patients selection

Out of 35 patients with interstitial lung disease (ILD) who underwent TBLC between January 2018 and August 2018 from HARUHI Respiratory Medical Hospital, Kiyosu, Japan, seven were identified to receive SLB. All patients had clinical and radiological features consistent with fibrotic ILD, but CT showed patterns of indeterminate for UIP or alternative diagnosis for UIP based on the official ATS/ERS/JRS/ALAT clinical practice guideline of idiopathic pulmonary fibrosis [18]. We carried out TBLC on these patients but were unable to make a diagnosis, therefore we proceeded to perform SLB.

### TBLC

The procedure was performed under deep sedation with intravenous propofol and remifentanil. Patients were intubated with a flexible tracheoscope (BF-1TQ290 and BF-260, OLYMPUS, Japan), and the cryobiopsies were obtained using a flexible cryoprobe measuring 115 cm in length and 1.9 mm in diameter (ERBECRYO2, ERBE, Germany). The biopsies were obtained under fluoroscopic guidance using the flexible bronchoscope inserted through the orotracheal intubation tube. Particular caution was exercised with respect to the position of the biopsy: the cryoprobe was placed perpendicular to the chest wall to assure an accurate evaluation of the distance from the thoracic wall by fluoroscopy. A distance of approximately 10 mm from the thoracic wall was considered optimal. The biopsy site was decided by the bronchoscopist taking into consideration images obtained by HRCT scanning for each case. Once brought into position, the probe was cooled for approximately 5–6 s, then the frozen lung tissue attached on the probe tip was retracted. The frozen specimen was thawed in saline and then transferred to formalin for fixation. In case of bleeding, the site was compressed and suctioned through the tracheoscope. If bleeding continued, cold physiological saline, adrenaline or thrombin was sprayed through the tracheoscope. Within 2 h after the procedure, a chest X-ray was obtained to exclude pneumothorax. Oxygen was administered continuously through the rigid bronchoscope and spontaneous breathing was maintained during the procedure. Oxygen saturation, blood pressure, and ECG were monitored continuously.

### Pathologic assessment

Four pathologists (JF, KK, KT, and YZ) examined the pathologic specimens independently, recorded their individual impressions in a blinded fashion, and discussed among themselves to reach one final pathology interpretation for each case. Pathologists recorded their final diagnostic impression, their subjective confidence level (high or low), and the histological features observed which classified from 0 to 3 based on the severity. Histological features were evaluated in four stages from 0 to 3. Final interpretation of each case was reached by agreeing on the most likely diagnosis and on their global confidence level. TBLC was considered nondiagnostic when histopathologic criteria sufficient to define a characteristic histopathologic pattern were lacking. The official 2018 ATS/ERS/JRS/ALAT clinical practice guidelines for diagnosis of idiopathic pulmonary fibrosis were applied for histological diagnosis [18]. Original diagnosis, UIP guideline diagnosis, and etiological diagnosis based on multidisciplinary discussion with expert pulmonologists and radiologists were obtained for all cases.

## Results

### Patient background

Clinical information and TBLC status of the 7 cases are presented in Table 1. Subjects included 5 males and 2 females, and their median age was 75 years. In all cases, the disease course was chronic. HRCT showed indeterminate for UIP pattern in 2 cases and alternative Diagnosis pattern in 5 cases. Approximately 3–6 TBLC specimen were taken in each case. The median size of TBLC specimen were 4.5–8.5 mm which was sufficient for pathological diagnosis. Except for 1 case, SLB was performed due to low confidence level of TBLC diagnosis. The median duration between TBLC and SLB was 30 days. Regarding adverse events due to TBLC, 2 cases showed sustained airway bleeding which continued after the examination. Pneumothorax, pneumomediastinum, respiratory failure, and acute exacerbation of ILD accompanying the examination were not observed. There was no adverse event after SLB.

**Table 1:** Clinical data, HRCT pattern of IPF guideline Diagnosis, and TBLC status of all 7 cases

Case No.	Age	Sex	Smoking (py)	HRCT pattern of IPF guideline Dx	Number of TBLC samples	Size of TBLC specimen (mm, mean value)	TBLC Dx confidence level	Duration between TBLC date and SLB date (day)
1	78	F	0	alternative Dx	6	4.95	low confidence	22
2	71	F	13	alternative Dx	6	5.97	low confidence	38
3	79	M	75	alternative Dx	3	7.66	low confidence	34
4	54	M	0	indeterminate for UIP	5	4.54	high confidence	86
5	56	M	35	indeterminate for UIP	4	8.53	low confidence	30
6	75	M	10	alternative Dx	6	5.44	low confidence	30
7	77	M	0	indeterminate for UIP	3	5.46	low confidence	23

*HRCT* high resolution computed tomography, *TBLC* transbronchial cryobiopsy, *SLB* surgical lung biopsy, *Dx* diagnosis, *UIP* usual interstitial pneumonia

### Pathological diagnosis of TBLC and SLB

**Fig. 1:**
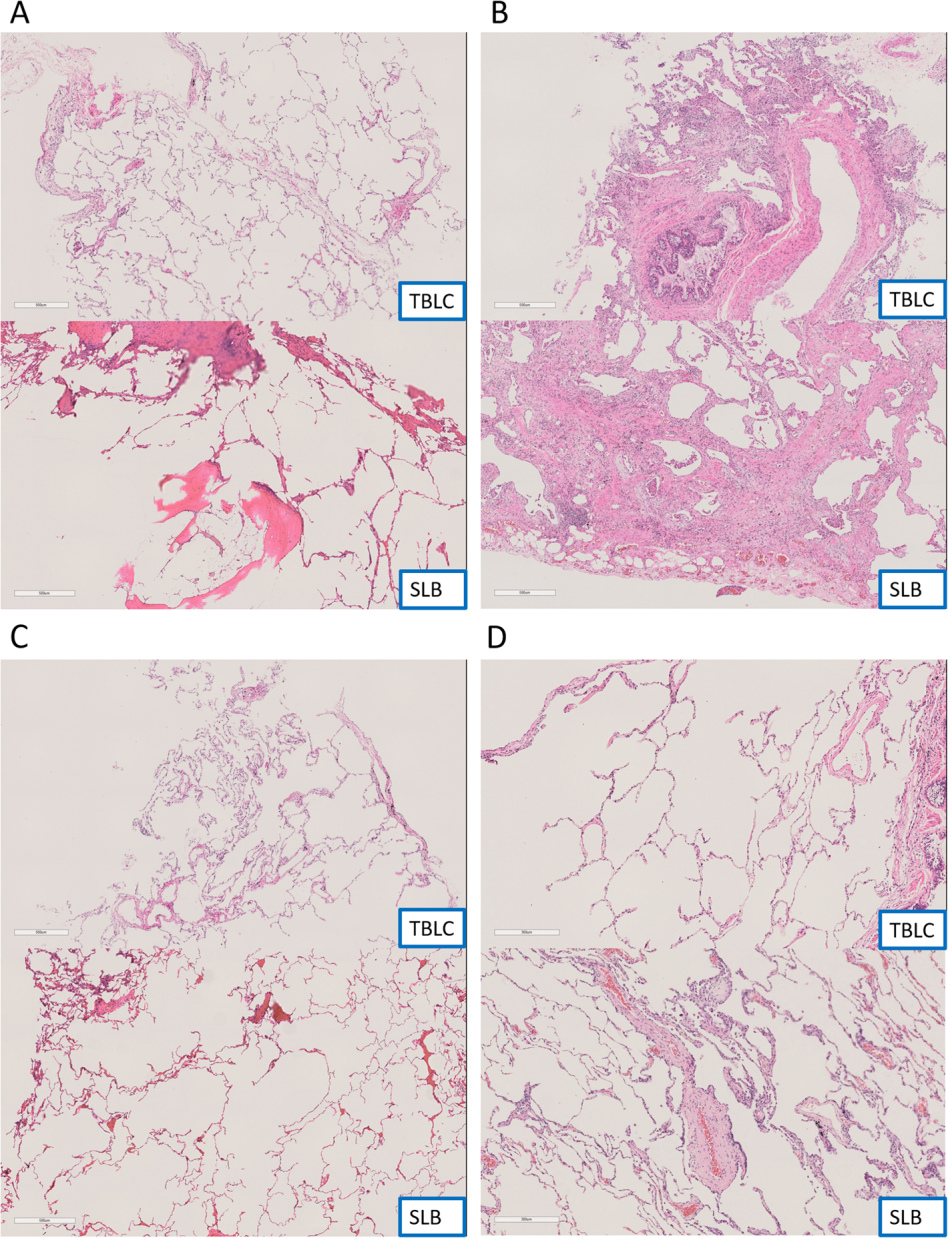
**a**: TBLC specimen shows mild invasion of inflammatory cells in the alveolar septum. SLB specimen reveals bone formation with myeloid tissue along the interstitium. **b**: Dense fibrosis and fibroblastic foci found in TBLC specimen were also seen in SLB specimen. **c**: Cellular IP shown in TBLC specimen were less represented in SLB specimen. **d**: Organizing pneumonia and PBM seen in SLB specimen were not noted in TBLC specimen

Table 2 summarizes the pathological diagnosis of TBLC and SLB for all 7 cases. Pathological diagnosis and multidisciplinary diagnosis were possible in all cases. Pathological diagnosis based on the UIP guideline with TBLC was 1 definite UIP pattern, 1 probable UIP pattern, 2 indeterminate for UIP pattern, and 3 alternative diagnosis patterns. Pathological diagnosis with TBLC and SLB had agreement in 5 cases, and the diagnosis was changed from indeterminate for UIP pattern with TBLC to probable UIP with SLB in the remaining 2 cases. In 3 cases which were diagnosed as alternative diagnosis pattern by TBLC, one case was diagnosed as NSIP by TBLC and SLB, but the other 2 cases resulted in different diagnosis between TBLC and SLB. Estimated etiology based on multidisciplinary diagnosis were consistent between the diagnosis with TBLC and SLB. The other 2 cases were diagnosed as alternative diagnosis pattern based on the UIP guideline. In 1 case (Case 6), NSIP was suspected with TBLC as inflammation was observed only in the alveolar septum; however, bone formation along the interstitium with only slight inflammation of the alveolar septum was observed with SLB, which resulted in changing the diagnosis to dendriform pulmonary ossification (Fig. 1a).

**Table 2:** Pathological diagnosis in all 7 cases

	Pathological Diagnosis		UIP guideline Diagnosis		Etiology TBLC+MDD		Etiology SLB + MDD	
Case No.	TBLC	SLB	TBLC	SLB	1st	2nd	1st	2nd
1	probable UIP	probable UIP	probable	probable	CHP	IPF	IPF	
2	cellular and fibrotic IP	probable UIP	indeterminate	probable	CHP	IPF	CHP	IPF
3	Fibrotic IP with bronchiolitis	ACIF	alternative	alternative	CHP	SR-ILD	SR-ILD	CHP
4	definite UIP	definite UIP	definite	definite	IPF		IPF	
5	fibrotic IP with DIP reaction	UIP and NSIP	indeterminate	probable	IPF		IPF	
6	Cellular IP, NOS	DPO	alternative	alternative	UCIP	iNSIP	idiopathic DPO	
7	cellular and fibrotic IP favor NSIP	NSIP with OP	alternative	alternative	NSIP with AE	IPF with AE	UCIP	chronic ASS

*TBLC* transbronchial cryobiopsy, *SLB* surgical lung biopsy, *MDD* multidisciplinary discussion, *IP* interstitial pneumonia, *UIP* usual interstitial pneumonia, *NSIP* nonspecific interstitial pneumonia, *NOS* not otherwise specified, *OP* organizing pneumonia, *DIP* desquamative interstitial pneumonia, *ACIF* airway centered interstitial fibrosis, *DP*O dendriform pulmonary ossification, *IPF* idiopathic pulmonary fibrosis, *CHP* chronic hypersensitivity pneumonia, *iNSIP* idiopathic nonspecific interstitial pneumonia, *SR-ILD* smoking related interstitial lung disease, *UCIP* unclassifiable interstitial pneumonia, *AE* acute exacerbation, *ASS* antisynthetase syndrome

### Pathological findings

Table 3 summarizes the pathological findings of TBLC and SLB for all 7 cases. Dense fibrosis, peripheral distribution, and fibroblast foci that are important histological findings in UIP guideline-based pathological diagnosis were mostly consistent between TBLC and SLB (Fig. 1b). Honeycombing was difficult to identify with TBLC compared to SLB. Diffusely spreading lesions such as cellular IP tended to be overestimated with TBLC compared to SLB (Fig. 1c). Lesions such as organizing pneumonia, airway lesions, and peribronchiolar metaplasia which are scattered in the lung were sometimes not included in the collected TBLC sample and were therefore unable to be diagnosed (Fig. 1d).

**Table 3:**
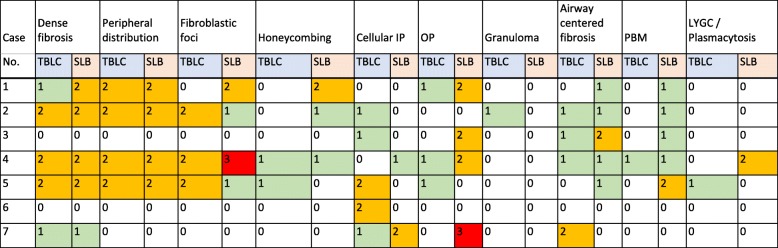
Pathological findings in all 7 cases

0, none; 1, mild; 2, moderate; 3, severe

IP: interstitial pneumonia, OP: organizing pneumonia, PBM: peribronchiolar metaplasia, LYGC: lymphoid follicles with germinal center

## Discussion

TBLC has been reported to be useful for the diagnosis of diffuse interstitial lung disease and its use has been increasing since 2014 [19]. However, no study has compared the histopathological findings and diagnosis of the same patient using TBLC and SLB. This is the first report in which 7 patients with diffuse pulmonary disease who underwent both TBLC and SLB were selected and their histopathological findings as well as diagnosis were compared.

TBLC and SLB diagnosis based on UIP guideline were consistent in 5 out of 7 cases. In the 2 cases with discordant results, the original diagnosis with TBLC was not contradicted but rather changed from indeterminate for UIP to probable UIP. There was no false positive diagnosis of UIP with TBLC. Although the specificity of TBLC may be inferior in UIP diagnosis, its sensitivity is determined to be satisfactory. In other words, if enough observational findings that indicate UIP are obtained using TBLC, a definitive diagnosis of UIP without SLB could possibly be provided.

Histopathological assessment included examination of 10 items. Dense fibrosis, peripheral distribution, and fibroblastic foci which are the diagnostic basis of UIP according to the official 2018 ATS/ERS/JRS/ALAT clinical practice guidelines for diagnosis of idiopathic pulmonary fibrosis [18] were largely consistent between TBLC and SLB diagnoses., discordant tendencies were noted in the assessment of diffuse lesions such as cellular IP, lesions around the airways such as PBM, and discrete lesions of the lung such as organized pneumonia. This may be causally related to the attributes of TBLC; disseminated lesions within the lung may not be collected using this method, fibrosis along the peripheral lung or other main lesions of the patient may not be represented in the collect specimen, and the small sample may over assess regions that are considered minor in SLB specimen. Pathologists must be aware of these biases which could lead to misdiagnosis. Because specimen do not contain tissues directly beneath the pleura, all 4 pathologists participating in this study agreed that lung tissue around the bronchovascular bundle should be recognized as peripheral lobule and make a decision based on their assessment.

Tomasetti et al. suggested that MDD improves the accuracy of IPF diagnosis with cryobiopsy, and therefore it is a critical element to diagnosis [15]. There was 1 case in our study which was difficult to be determined as IPF with cryobiopsy only and after MDD, CHP was more strongly suspected (case 2). MDD is thought to be highly significant in diagnosing ILD with TBLC.

**Fig. 2:**
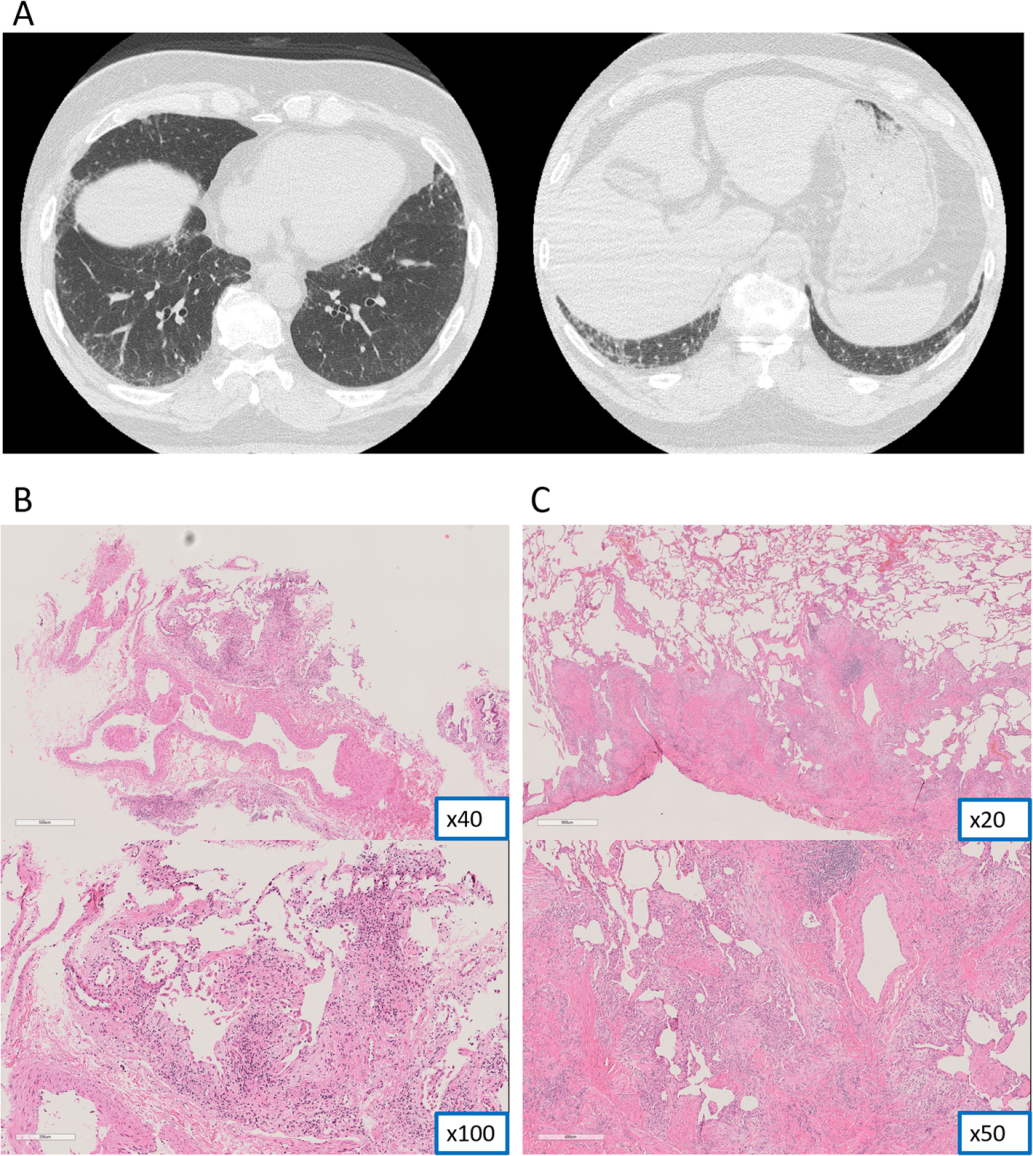
**a**: HRCT shows linear and ground-glass opacities at the base of the lung. **b**: TBLC specimen indicates dense fibrosis with structural modifications in the edge of pulmonary lobule as well as fibroblast foci. **c**: SLB specimen reveals dense fibrosis and fibroblast foci with structural modifications spreading at the subpleural region and near the intralobular septum

SLB is generally performed in cases with low diagnostic confidence of cryobiopsy, which includes not only the uncertainty of histopathological diagnosis but also the inconsistency with HRCT and clinical findings. A case was encountered in this study in which UIP diagnosis with high confidence level was provided but SLB was also performed. The HRCT result of this case showed indeterminate for UIP pattern, and the patient was too young for IPF to be suspected [20, 21] and these inconsistencies resulted in additional SLB (Fig. 2).

Recently, Romagnoli et al. reported poor concordance of pathology diagnoses between TBLC and SLB [17]. Careful evaluation of the contents revealed there were nine UIP diagnosis with TBLC among which seven were also considered to have UIP pattern with SLB. Within the seven UIP pattern diagnoses with SLB, two were favored more as CHP over IPF by pathology, however, after multidisciplinary diagnosis, both cases were concluded to be IPF. Eventually, out of the nine cases of UIP with TBLC, six were diagnosed as IPF after multidisciplinary discussion. Their result is somewhat similar to our series in terms of having higher agreement in UIP judgement than other histologic patterns.

This study has some limitations. In particular, this was a single facility pathological study including only a small number of cases which were determined by MDD that TBLC was insufficient to make a definite diagnosis. Only seven cases in which patients underwent both TBLC and SLB were selected. Additional SLB is not necessary if the confidence level of TBLC diagnosis is high, so only those with low confidence TBLC diagnosis or whose TBLC diagnosis was inconsistent with clinical or imaging findings underwent SLB. Therefore, this may have some biases and many not truly reflect a true comparison of TBLC and SLB. A higher concordance is expected by comparing pathological findings and diagnosis in cases with highly confident TBLC diagnosis. Iftikhar IH et al. shows that pooled diagnostic yield, pooled sensitivity, and pooled specificity of TBLC were 83.7, 87, and 57%, respectively [22]. However, this report was a systematic review of past literature, and no document which examined TBLB and SLB in the same case was included. In terms of standardization of definitive diagnosis, it is important to collect cases from multiple facilities and study their concordance of histopathological assessments and diagnoses. Additionally, it is vital to collect evidence in terms of indication for TBLC and the need for additional SLB in order to develop diagnostic guidelines.

## Conclusions

This study showed that TBLC is somewhat inferior in sensitivity yet relatively high in specificity for diagnosing UIP. It is suggested that if enough observational data is obtained from TBLC to determine UIP, definitive diagnosis could be possible without SLB. Meanwhile, additional SLB may be indicated for the diagnosis of other ILD such as NSIP and SR-ILD, since TBLC may be inferior in specificity in these cases.

## Data Availability

Data and materials of this work are available from the corresponding author upon reasonable request.

## Abbreviations

CHP: Chronic hypersensitivity pneumonitis
CT: Computed tomography
ECG: Electrical cardiogram
HRCT: High resolution computed tomography
ILD: Interstitial lung disease
IP: Interstitial pneumonia
IPF: Idiopathic pulmonary fibrosis
MDD: Multidisciplinary diagnosis
NSIP: Non-specific interstitial pneumonia
PBM: Peribronchiolar metaplasia
SLB: Surgical lung biopsy
SR-ILD: Smoking related interstitial lung disease
TBLC: Transbronchial lung cryobiopsy
UIP: Usual interstitial pneumonia
